# Spatial distribution characteristics and accessibility analysis in the Weibei Imperial Mausoleum Protection Zone under the hierarchical clustering algorithm

**DOI:** 10.1371/journal.pone.0321847

**Published:** 2025-05-08

**Authors:** Xiaojie Feng

**Affiliations:** Civil & Architecture Engineering Institute, Xi’an Technological University, Xi’an, China; Humboldt-Universitat zu Berlin, GERMANY

## Abstract

This study mainly aims to investigate the spatial distribution characteristics and accessibility of the Weibei Imperial Mausoleum Protection Zone, and gain a deeper understanding of cultural heritage preservation and urban planning in this area. Settlements represent areas where people reside over the long term, including villages, towns, etc., and their spatial distribution reflects the interplay between human activities and the natural environment, as well as historical heritage. The relationship among population distribution, land use, cultural heritage preservation areas, and resident lifestyles can be understood by analyzing the spatial distribution of settlements. This is crucial for formulating effective urban planning and cultural heritage protection strategies. Initially, factor analysis is employed to reduce the dimensionality of the original multidimensional data. Subsequently, the hierarchical clustering algorithm is applied to categorize and cluster settlements based on these factors, then combines them into clusters, thereby revealing the spatial distribution patterns among different settlements. Next, this study constructs a settlement spatial distribution model based on factor analysis combined with the hierarchical clustering algorithm. Additionally, through accessibility analysis, this study examines the situation of residents accessing cultural heritage sites at different times during weekdays and weekends. The results indicate that with a classification number of 4, the Goodness of Variance Fit (GVF) for different hierarchical features exceeds 0.7, and the average silhouette coefficient reaches 0.58 at this classification number. Furthermore, the accessibility analysis also illustrates residents’ visiting patterns to cultural heritage sites on weekends and weekdays, providing valuable insights for urban planning and cultural heritage preservation. Therefore, this study concludes that categorizing settlements into four classes in this area is reasonable and emphasizes the importance of cultural heritage site preservation and urban planning. This method demonstrates a high level of accuracy and interpretability in spatial analysis.

## Introduction

With the continuous global focus on cultural heritage protection and urban planning, the Weibei Imperial Mausoleum Protection Zone in China offers a unique case study. This protection zone, located north of the Wei River basin in Shaanxi Province, China, has a rich history and culture, boasting numerous imperial tombs, ancient architecture, and a wealth of cultural heritage [1,2]. These relics are not only crucial for understanding the development of ancient Chinese history and culture but also significantly contribute to the diversity and preservation efforts of global cultural heritage. However, with the rapid advancement of urbanization and the swift development of the tourism industry, the protection and management of this zone face increasingly complex challenges [3]. The Weibei Imperial Mausoleum Protection Zone may not be as internationally renowned as other famous cultural heritage sites in China, such as the Great Wall or the Forbidden City. However, its unique historical value and the protection challenges it faces make it a field worthy of in-depth study [4]. This study aims to provide a scientific basis for cultural heritage protection and urban planning by analyzing the spatial distribution characteristics and accessibility of settlements within the protection zone. Meanwhile, it also reveals the importance and complexity of the zone to an international audience.

The importance of cultural heritage protection and urban planning has been widely recognized worldwide. For example, Wang et al. investigated the spatial distribution characteristics and influencing factors of intangible cultural heritage in the Yellow River Basin [5]. Li et al. identified the influencing factors and spatial distribution of cultural heritage among ethnic minorities in Southwest China [6]. However, there remains a research and practical gap in effectively protecting and managing cultural heritage sites in the context of rapid urbanization and tourism development. This challenge is particularly pronounced in the Weibei Imperial Mausoleum Protection Zone in Shaanxi Province, China. The Weibei Imperial Mausoleum Protection Zone is not only a significant historical and cultural heritage site in China but also a valuable resource for studying ancient tomb architecture, sculptural art, and funeral customs. However, the spatial distribution characteristics and accessibility of cultural heritage sites in this zone have not been fully studied, limiting a comprehensive understanding of the potential for cultural heritage protection and utilization in the zone. Moreover, existing studies have mostly focused on the spatial distribution analysis of single cultural heritage sites or small-scale areas, with relatively insufficient research on large-scale or multi-scale spatial distribution characteristics. At the same time, most studies emphasize descriptive analysis of spatial distribution characteristics, with little consideration for the practical applications of cultural heritage protection and management.

Protecting and maintaining cultural heritage is a shared responsibility of governments and societies worldwide. The Weibei Imperial Mausoleum Protection Zone, an integral part of China’s cultural heritage, represents a rich historical legacy and significant cultural significance [7]. As a powerful data mining tool, the hierarchical clustering algorithm can categorize and group cultural heritage points within the Weibei Imperial Mausoleum Protection Zone [8]. This algorithm can help identify similar cultural heritage points in space and reveal their associations. By applying the hierarchical clustering algorithm, the interrelationships among various cultural heritage points within the area and their spatial distribution patterns can be better understood [9,10]. This contributes to offering more effective management recommendations for preserving cultural sites, striking a balance between visitor flow and the preservation needs of cultural heritage.

This study aims to provide an in-depth analysis and understanding of the spatial distribution and accessibility of cultural heritage sites. Moreover, it offers scientific evidence and practical guidance for the cultural heritage protection, urban planning, and sustainable development of the Weibei Imperial Mausoleum Protection Zone. By applying hierarchical clustering algorithms and factor analysis methods, this study innovatively focuses on the spatial distribution characteristics of cultural heritage within the Weibei Imperial Mausoleum Protection Zone. Additionally, combined with protection and management requirements, a new model is proposed. This model enhances the accuracy and interpretability of spatial analysis. Meanwhile, it provides strong support for the optimization of spatial layout in cultural heritage areas, offering new perspectives and methods for cultural heritage protection and urban planning.

The overall organizational structure is as follows. Section 1 is the introduction, which introduces the background and importance of the study, and expounds the necessity of the spatial distribution characteristics and accessibility analysis of cultural heritage in Weibei Imperial Mausoleum Protection Zone. Section 2 is the literature review, which summarizes and analyzes the research status of quantification of settlement spatial characteristics and the application of a hierarchical clustering algorithm in spatial data analysis. Section 3 describes the research methods in detail, including data collection, the hierarchical clustering algorithm and its improved analysis, and the settlement spatial distribution model based on factor analysis and hierarchical clustering algorithms. Section 4 presents the research results, encompassing the classification of spatial distribution characteristics, the analysis of Goodness of Variance Fit (GVF) and silhouette coefficients, as well as the accessibility patterns of residents’ access to cultural heritage sites on weekdays and weekends. Section 5 summarizes this study’s main findings, puts forward suggestions for optimizing the spatial layout of cultural heritage areas, points out the study’s limitations, and proposes prospects for future research directions.

## Literature review

### The current state of research on the quantification of settlement spatial characteristics

The quantification of settlement spatial characteristics employs quantitative methods and statistical analysis to measure and analyze the distribution, density, morphology, and other relevant attributes of settlements (such as villages and towns) in geographical space. It aims to better understand and interpret the relationships between these settlements and the natural environment, socio-economic factors, and cultural heritage [11]. The quantification of settlement spatial characteristics is not limited to the cultural heritage field. It has a wide range of applications in various fields, including urban planning, environmental conservation, and natural disaster management. Numerous scholars conducted research in this area. In the Geographic Information System (GIS), the unity of spatial resolution and scale of data is the foundation for accurate spatial analysis. For example, Qi et al. using GIS analysis and regression models, explored the spatial distribution characteristics of rural tourism villages on the Qinghai-Tibet Plateau and their influencing factors. The results indicated that the distribution of rural tourism villages was significantly influenced by natural factors such as altitude, temperature, and precipitation [12]. Bian et al. studied the spatial distribution characteristics and influencing factors of traditional villages in China using GIS and statistical analysis. The findings suggested correlations between village distribution and geographical location, historical and cultural factors, and the degree of urbanization [13]. Xu et al. investigated the geographic distribution characteristics of ethnic minority villages in Fujian Province and their relationship with topographical factors. They used GIS and regression analysis to reveal associations between topographical factors like mountains and water bodies and the distribution of ethnic minority villages [14]. Xiang et al. quantitatively analyzed the spatial distribution and connectivity of chieftain’s site relics. They employed network analysis methods to uncover spatial relationships among chieftain’s sites, contributing to a better understanding of the historical geographic patterns of these cultural heritage sites [15]. Zhou et al. researched the cultural landscape perception of traditional Chinese settlements by analyzing online visitor reviews. The findings showed that visitor comments could provide insights into the perception and values associated with cultural heritage sites, offering valuable information for planning and promoting cultural heritage tourism [16].

Zhang et al. combined repeated Lidar with Landsat products to quantify permafrost melting subsidence caused by fires in the interior of Alaska. The results provided a new method for large-scale assessment of the impact of fires on permafrost regions [17]. Lin et al. investigated the spatial form evolution of traditional villages in Jiuguan under the influence of a historical traffic network. Through the quantitative analysis of settlement space, the far-reaching impact of traffic networks on village structure and development was revealed, which offered a vital reference for cultural heritage protection [18]. He et al. proposed a settlement prediction method for immersed tube tunnels by considering the time-dependent foundation modulus. This study, integrated with engineering practice, furnished an in-depth understanding of the stability of cultural heritage foundations and contributed to protecting cultural heritage in tunnel construction and maintenance [19].

Additionally, many scholars conducted related research on cultural heritage. Kuang et al. analyzed the spatial distribution characteristics and influencing factors of intangible cultural heritage (ICH) in central China. They revealed the impact of geographical location, economic development, and historical background on the distribution of cultural heritage, which helped formulate protection strategies [20]. Nie et al. discussed the spatial distribution and influencing factors of Chinese traditional ICH drugs. They clarified the distribution pattern of these heritages by analyzing geographical and socioeconomic factors and provided a scientific basis for protection and inheritance [21]. Zhang et al. analyzed the spatial distribution of China’s ICH resources and its influencing factors. They demonstrated the impact of natural geographical conditions, socio-economic development, and policy support on the distribution of cultural heritage, which was conducive to more effective protection and utilization of cultural resources [22].

### The current application status of the hierarchical clustering algorithm

The hierarchical clustering algorithm is a layer-based clustering process that divides data into multiple layers, each containing clusters of different granularity [23]. This makes it suitable for analyzing spatial data with multi-level relationships. Numerous scholars investigated the current application status of hierarchical clustering algorithms. For instance, Bin and Sun used a multi-functional complex network model to study the problem of influence maximization in social networks. The results revealed that this model could be used to understand information dissemination patterns in social networks, contributing to the analysis of information spread in space [24]. Wang and Lv constructed a personalized chemistry major learning and knowledge system based on the Internet of Things and clustering algorithms. This system could inspire spatial distribution analysis, facilitating the study of the distribution of cultural heritage sites [25]. Liu et al. proposed a hierarchical clustering-based dynamic load identification model and applied it to mechanical systems. The hierarchical clustering method could be used in geographic spatial analysis to study the spatial relationships of locations [26]. Zerouali et al. utilized wavelet transform information and hierarchical clustering analysis to explore rainfall patterns in northeast Algiers. This method could be used for spatial distribution analysis of regional climate patterns [27]. Ran et al. provided a comprehensive review of hierarchical clustering algorithms and their latest developments, offering a comprehensive understanding of hierarchical clustering methods in spatial distribution research [28]. El-Rawy et al. evaluated groundwater quality in the Jazan region of Saudi Arabia using principal component analysis (PCA) and hierarchical clustering analysis. This approach could be used in spatial distribution research to analyze the distribution of groundwater resources [29]. Huang et al. introduced an integrated hierarchical clustering algorithm combining the advantages of cluster and partition level to improve the clustering quality of spatial data in multi-level relationships. The results revealed that the algorithm performed well in processing complex datasets [23]. Moseley & Wang discussed the approximate bounds of hierarchical clustering algorithms (average linkage, bisecting k-means, and local search), verified these algorithms’ effectiveness in multi-level relational spatial data, and provided a theoretical basis for algorithm selection [30]. Rizalde et al. compared the performance of K-means, BIRCH, and hierarchical clustering algorithms in the data clustering of obsessive-compulsive symptoms. The findings denoted that hierarchical clustering had unique advantages in processing multi-level relationships and high-dimensional spatial data [31]. Misra et al. analyzed the performance of hierarchical clustering applied in multi-user MIMO NOMA networks. They found that this method effectively processed multi-level relational data in the network and improved the system’s overall performance and resource allocation efficiency [32].

### Summary

Through the analysis of existing literature, it has been found that hierarchical clustering algorithms are widely applied in the field of cultural heritage studies due to their ability to handle multi-scale spatial data. However, existing studies mostly focus on single cultural heritage sites or small-scale areas, with relatively insufficient research on large-scale or multi-scale spatial distribution characteristics. Moreover, most studies highlight descriptive analysis of spatial distribution characteristics and seldom involve the practical applications of cultural heritage protection and management. Hence, this study innovatively focuses on the spatial distribution characteristics of cultural heritage within the Weibei Imperial Mausoleum Protection Zone by combining factor analysis and hierarchical clustering algorithms. It constructs an integrated model to assess the spatial distribution characteristics of cultural heritage in this zone, enhancing its accuracy and interpretability of spatial analysis. The model reveals the spatial hierarchical relationships between different settlements. Additionally, it provides a new perspective for dynamic traffic management and the sustainable use of cultural heritage through accessibility analysis that includes temporal factors.

## The spatial distribution and accessibility of rural settlements in the Weibei Imperial Mausoleum Protection Zone

This study employs an innovative methodology to deepen the spatial distribution characteristics and accessibility analysis of settlements in the Weibei Imperial Mausoleum Protection Zone by combining a hierarchical clustering algorithm and factor analysis. This method not only effectively reduces the dimension of the original data and extracts the key factors but also reveals the spatial hierarchy of the settlements through hierarchical clustering. In addition, for the first time, the study includes time factors in accessibility analysis, examining access patterns over different periods, and offering new perspectives for dynamic traffic management and sustainable use of cultural heritage. Constructing the comprehensive evaluation model enhances the accuracy and interpretability of the analysis. Furthermore, it provides an innovative decision-support tool for the spatial planning and management of cultural heritage areas.

### Data collection in the Weibei Imperial Mausoleum Protection Zone

**Fig 1:**
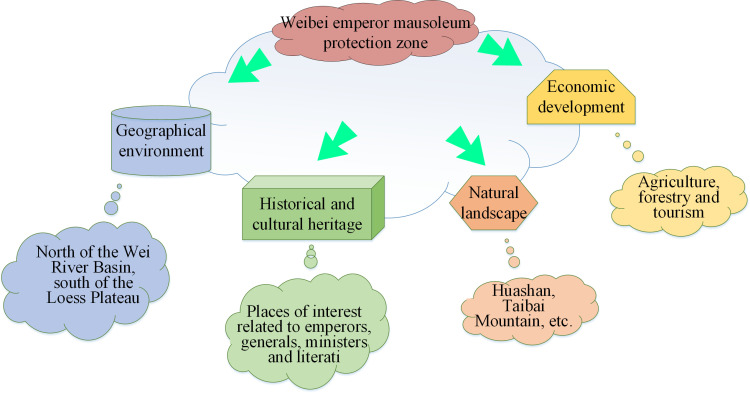
Schematic map of the characteristics of the Weibei Imperial Mausoleum Protection Zone.

The term Weibei Imperial Mausoleum Protection primarily refers to the area encompassing important historical and cultural heritage sites such as the Mausoleum of the First Qin Emperor and the Han and Tang Imperial Mausoleums, located north of the Wei River Basin in Shaanxi Province, China. This area is rich in natural and cultural resources, making spatial analysis of its settlements of significant historical importance. It is also significant for preserving and developing cultural heritage and traditional Chinese culture [33,34]. Fig 1 presents the characteristics of the Weibei Imperial Mausoleum Protection Zone.

Fig 1 presents the characteristics of the Weibei Imperial Mausoleum Protection Zone from four aspects: geographical environment, historical and cultural heritage, natural landscape, and economic development. Regarding the geographical environment, the Weibei Imperial Mausoleum is located north of the Wei River Basin in Shaanxi Province, in the southern part of the Loess Plateau. The area features diverse topography, including mountains, hills, plateaus, and plains. The Weibei Imperial Mausoleum Protection Zone is a temperate continental climate with four distinct seasons, with hot summers and cold winters. Considering historical and cultural heritage, the area has a rich historical legacy, with numerous emperors, statesmen, literary figures, and renowned historical sites contributing to its cultural wealth. In the realm of natural landscapes, the area boasts picturesque features such as towering peaks, meandering streams, and lush forests. Additionally, it is home to several famous natural attractions like Huashan and Taibai Mountain, which attract a large number of tourists. Regarding economic development, the primary industries include agriculture, forestry, and tourism [35].

### Hierarchical clustering algorithm and its improvement analysis

**Fig 2:**
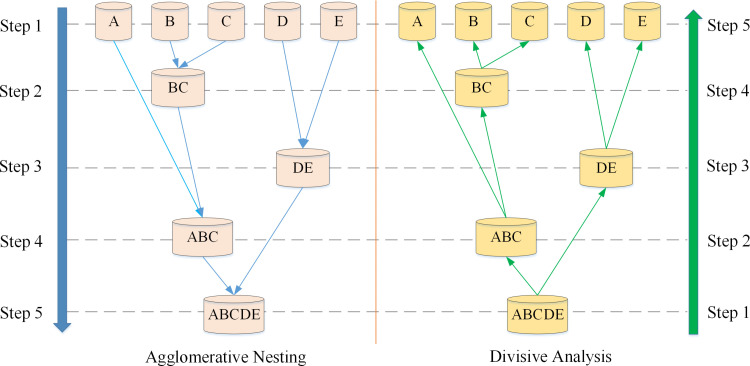
The schematic diagram of the hierarchical clustering algorithm.

When conducting spatial distribution analysis in cultural heritage areas, the hierarchical clustering algorithm can automatically discover potential spatial distribution patterns in the data without requiring prior assumptions [28]. This gives it a unique advantage in cultural heritage research. Depending on the partitioning strategy, hierarchical clustering algorithms can be divided into two types: Agglomerative Nesting (AGNES) and Divisive Analysis (DIANA) algorithms [36], as displayed in Fig 2.

Fig 2 illustrates the processing of two partitioning strategies. The agglomerative hierarchical clustering algorithm adopts a top-down approach. It initially treats each object as a cluster and gradually merges them according to certain criteria until all objects are in one cluster or meet some termination condition. Besides, divisive hierarchical clustering employs a bottom-up strategy, initially placing all objects in a single cluster and then gradually splitting them based on certain criteria until each object forms its cluster or meets some termination condition.

For the basic hierarchical clustering algorithm, decisions that have been made cannot be undone when making choices for merging or splitting points, which can easily lead to low-quality clustering results. Additionally, it is unsuitable for large datasets due to its high computational and spatial complexity. The Clustering Using Representative (CURE) algorithm is an improved version of agglomerative hierarchical clustering, designed to identify clusters with complex shapes and varying sizes while effectively reducing the impact of noise on clustering [37].

In this algorithm, the indicator value for rural settlement spatial features is set as $x_{ci}\left(i=0,1,\cdots ,p_{c}-1\right)$ . $p_{c}$ represents the number of indicators, and a new variable $x_{ci}$ is obtained by normalizing $x_{ci}$, which together form the original dataset $S_{c}$. $z_{ci}$ can be seen as individual data points. First, a portion of $z_{ci}$ is randomly selected from $S_{c}$ as a subset $S_{ci}$, and then this subset is partitioned. The CURE clustering algorithm is run on these partitioned sets to obtain clusters for each set and remove outliers. Finally, the remaining data points in the dataset $S_{c}$ are partitioned. Hence, the metric method of inter-cluster distance is adopted in this study. Inter-cluster distance refers to the distance between different clusters, usually defined as the distance between the two closest points in two clusters. This metric helps determine the boundaries of clusters and decide when to merge clusters during the clustering process. The inter-cluster distance falls between ward and average, and it is defined as the distance between the two closest representative points in two clusters, as indicated in equation (1):


$$dist\left(u,v\right)=\underset{z_{ci}\in u.rep,z_{ci}\in v.rep}{\min}\;dist\left(z_{ci},z_{cj}\right)$$ (1)

*u* and *v* refer to the set of points used in the clustering algorithm to represent their respective clusters. $dist\left(z_{ci},z_{cj}\right)$ represents the Lagrange distance between representative points $z_{ci},z_{cj}$.

When the algorithm is applied to extract spatial distribution features, the first step is from initializing parameters and selecting representative points to forming clusters and determining cluster boundaries. After that, the algorithm operates by randomly selecting subsets of data, performing partition clustering on the subsets, removing outliers, and assigning the remaining data points to corresponding clusters. It uses the nearest neighbor distance indicator to determine the distance between clusters, achieved by calculating the distance between the closest representative points of two clusters. This process not only reveals how the CURE algorithm effectively handles clusters of complex shapes and sizes but also demonstrates how it reduces the impact of noise on clustering results. Thus, it provides a powerful tool for spatial distribution analysis in the Weibei Imperial Mausoleum Protection Zone.

### Constructing a settlement spatial distribution model based on factor analysis integrated with the hierarchical clustering algorithm

**Fig 3:**
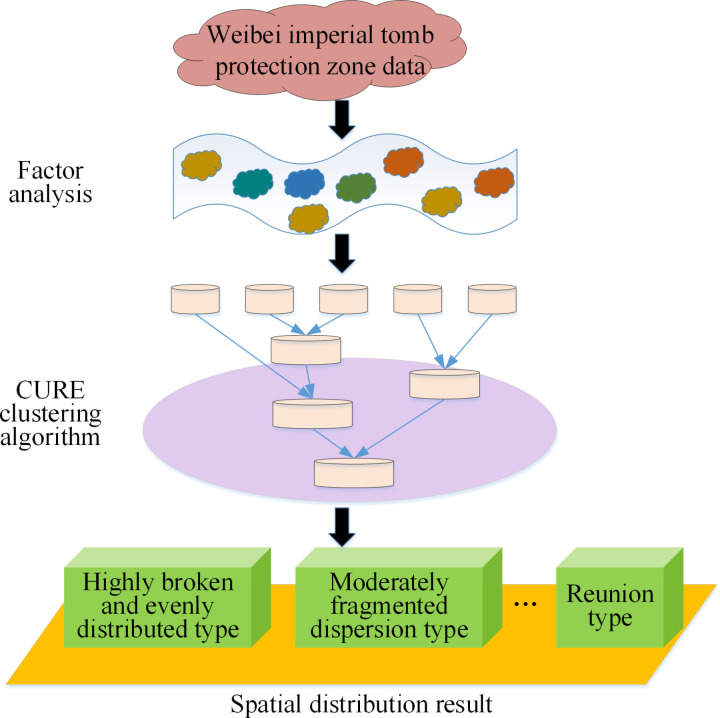
A schematic diagram of the settlement spatial distribution model based on factor analysis integrated with the hierarchical clustering algorithm.

Here, to effectively understand the spatial distribution of settlements in the Weibei Imperial Mausoleum Protection Zone, factor analysis is introduced to mitigate the dimensionality of the original multidimensional data, reduce information redundancy, and extract latent factors [38]. Additionally, the hierarchical clustering algorithm classifies and groups settlements using these factors. This helps identify settlements with similar characteristics and group them into clusters, revealing spatial distribution patterns among different settlements. Fig 3 underscores the settlement spatial distribution model constructed based on the integration of factor analysis with a hierarchical clustering algorithm.

In Fig 3, the model combines factor analysis and hierarchical clustering algorithms, first reducing the dimensionality of the original multi-dimensional data through factor analysis to extract key factors affecting settlement distribution. Then, these factors are used as inputs to the clustering algorithm to classify and cluster settlements. This method identifies groups of settlements with similar characteristics and reveals their spatial distribution relationships. The model’s output includes feature descriptions of each cluster, spatial distribution maps, and the interrelationships between clusters, offering an intuitive spatial analysis tool for cultural heritage conservation and urban planning.

This model’s specific spatial distribution characteristic process is as follows. The indicator value for rural settlement spatial characteristics is set to $x_{i}\left(i=0,1,\cdots ,p-1\right)$, that is, the original variable. *p* represents the number of indicators. By standardizing $x_{i}$, a new variable $z_{i}$ is obtained. Then, a factor analysis model can be established, as shown in equation (2):


$$z_{i}=a_{i0}F_{0}+a_{i1}F_{1}+\cdots +a_{im-1}F_{m-1}+c_{i}U_{i}\left(i=0,1,\cdots ,p-1\right)$$ (2)

$F_{j}\left(j=0,1,\cdots ,m-1\right)$ represents the common factors, appearing in the expression of each variable, and their meanings need to be interpreted based on the research context and the original variable meanings. $U_{i}\left(i=0,1,\cdots ,p-1\right)$ is solely related to variable $z_{i}$ and is referred to as the specific factor. Coefficient $a_{ij},c_{i}$ represents factor loadings, and $A=\left(a_{ij}\right)$ is the factor loading matrix. Equation (1) can be expressed in matrix form, as follows:


Z=AF+CU (3)

*C* is the variance matrix for a particular factor. The model aims to extract the key factors affecting the distribution of settlements through dimensionality reduction. Each factor can be expressed as equation (4):


$$\begin{array}{c} \left\{ \;\;\;\begin{array}{c} {}*35lZ={\left(z_{0},z_{1},\cdots ,z_{p-1}\right)}^{T}\\ F={\left(F_{0},F_{1},\cdots ,F_{m-1}\right)}^{T}\\ U={\left(U_{0},U_{1},\cdots ,U_{p-1}\right)}^{T}\\ A={\left(a_{ij}\right)}_{\left(p-1\right)\times \left(m-1\right)}\\ C=diag\left(c_{0},c_{1},\cdots ,c_{p-1}\right) \end{array}\right. \end{array}$$ (4)

For this, the model typically assumes the following: 1) The special factors are mutually independent and independent from all common factors. 2) Each common factor is an independent normally distributed random variable with a mean of 0 and a variance of 1, and the covariance matrix is an identity matrix.

The contribution of the *m* common factors to the variance of the i-th variable is referred to as the *i*-th communality, denoted as $h_{i}^{2}$, as illustrated in equation (5):


$$h_{i}^{2}=a_{i0}^{2}+a_{i1}^{2}+\cdots +a_{im-1}^{2}$$ (5)

The variance of the specific factor is referred to as the specific variance or specific value, denoted as $\sigma_{i}^{2}$. Consequently, the variance of the *i*-th variable can be decomposed as:


$$Varz_{i}=h_{i}^{2}+\sigma_{i}^{2},i=0,1,\cdots ,p-1$$ (6)

When estimating the factor loading matrix for the constructed model, the most commonly used method is PCA based on the principal component model, and the results are tested using the maximum likelihood method.

$\lambda_{0}\geq \lambda_{1}\geq \cdots \geq \lambda_{p-1}$ is the eigenvalue of the sample correlation matrix *R*, and $\eta_{0},\eta_{1},\cdots ,\eta_{p-1}$ is the corresponding standard orthogonalized eigenvectors. Further analysis uses PCA to estimate factor load matrix *A* and the maximum likelihood method to test the results. If *m < p*, the loading matrix *A* for principal component factor analysis of the sample correlation matrix *R* is given by equation (7):


$$A=\left(\sqrt{\lambda_{0}}\eta_{0},\sqrt{\lambda_{1}}\eta_{1},\ldots ,\sqrt{\lambda_{m-1}}\eta_{m-1}\right)$$ (7)

The variance of the specific factor is estimated using the diagonal elements of $R-AA^{T}$, denoted as specific variance $\sigma_{i}^{2}$, as illustrated in equation (8):


$$\sigma_{i}^{2}=1-\sum {\mathrm{\backslash nolimits}}_{j=0}^{m-1}a_{ij}^{2}$$ (8)

The residual matrix can be represented as $R-AA^{T}-Cov\left(U\right)$. Thereby, when $AA^{T}+Cov\left(U\right)$ is close to the correlation matrix *R*, it can be intuitively considered that the factor model provides a better data fit.

The maximum variance method [39] is used for orthogonal rotation of the factor loading matrix. The factor loading matrix obtained from equation (2) can be represented as equation (9):


$$A=\left(\begin{array}{ccc} a_{0,0} & \cdots & a_{0,m-1}\\ \vdots & \ddots & \vdots \\ a_{p-1,0} & \cdots & a_{p-1,m-1} \end{array}\right)$$ (9)

The common factor variance (contribution rate) for the variable $z_{i}$ is expressed as equation (10):


$$h_{i}^{2}=\sum {\mathrm{\backslash nolimits}}_{j=0}^{m-1}a_{ij}^{2}$$ (10)

V(A) represents the total variance of the factor loading matrix *A*, as shown in equation (11):


$$V\left(A\right)=\sum {\mathrm{\backslash nolimits}}_{j=0}^{m-1}V_{j}=\sum {\mathrm{\backslash nolimits}}_{j=0}^{m-1}\sum {\mathrm{\backslash nolimits}}_{i=0}^{p-1}\left(d_{ij}^{2}-{\bar{d}}_{j}\right)$$ (11)

$$\begin{array}{c} \left\{ \;\;\;\begin{array}{c} {}*35ld_{ij}^{}=\frac{a_{ij}}{h_{i}}\\ {\bar{d}}_{j}=\frac{1}{p}\sum {\mathrm{\backslash nolimits}}_{i=0}^{p-1}d_{ij}^{2} \end{array}\right.,j=0,1,\cdots ,m-1 \end{array}$$ (12)

When the absolute values of each column in the factor loading matrix *A* for each factor tend towards 0 or 1, the value of V(A) is larger, indicating that the corresponding common factors have a simple structure. Here, an orthogonal matrix Γ is introduced to maximize V(AΓ). When the number of common factors is 2, that is, m = 2, the orthogonal matrix can be written as equation (13):


$$\Gamma =\left(\begin{array}{cc} \cos\varphi & -\sin\varphi \\ \sin\varphi & \cos\varphi \end{array}\right)$$ (13)

φ represents the rotation angle that maximizes the total variance of AΓ. When implementing the algorithm, a lower limit for the total variance can be specified based on the actual situation, or the rotation can be stopped when the total variance changes little. Further, the values of the common factors are given, and the linear combination of the original variables is represented as the common factor. That is, the factor score function reads:


$$F_{j}=b_{j0}z_{0}+\cdots +b_{jp-1}z_{p-1},\;j=0,1,\cdots ,m-1$$ (14)

Furthermore, sensitivity analysis is conducted to explore the sensitivity of each feature indicator to the corresponding common factor. Based on the characteristics of the data sample, a *p*-dimensional unit space $\Omega_{p}=\left\{z|0\leq z_{i}\leq 1;i=0,1,\cdots ,p-1\right\}$ is defined as the spatial domain of the dependent variable. Then, the function $F=f\left(z_{0},z_{1},\cdots ,z_{p-1}\right)$ is decomposed as follows:


$$F=f\left(z_{0},z_{1},\cdots ,z_{p-1}\right)=f_{0}+\sum_{i=0}^{p-1}f_{i}\left(z_{i}\right)+\sum_{i<l}^{p-1}f_{il}\left(z_{i},z_{l}\right)+\cdots +f_{0,1,\cdots ,p-1}\left(z_{0},z_{1},\cdots ,z_{p-1}\right)$$ (15)

$f_{0}$ is a constant, $f_{i}$ is the mapping function of $z_{i}$, and $f_{il}$ is a function of $z_{i}$ and $z_{l}$. There are $2^{k}$ sub-items on the right side of the equation, with each decomposition term satisfying equation (16):


$$\int_{0}^{1}f_{i_{0},i_{1},\cdots ,i_{s}}\left(z_{i_{0}},z_{i_{1}},\cdots ,z_{i_{s}}\right)dz_{k}=0,\;k=i_{0},i_{1},\cdots ,i_{s}$$ (16)

### Experimental data

To analyze the spatial distribution characteristics and accessibility of this area, this study primarily utilizes remote sensing image data, rural park vector data, and population data. These data provide basic support for analyzing the spatial distribution characteristics and accessibility of the Wei Bei Emperor Mausoleum Protection Area. The high-resolution satellite image data comes from Resource Satellite-3, Gaofen-1, and Gaofen-2 satellites, collected in February 2018. The data has high spatial resolution and can accurately identify surface features, especially in cultural heritage areas and human activity zones. In order to ensure data quality, all image data has undergone pre-processing such as radiometric correction, atmospheric correction, and geometric correction. Then, surface features are extracted through image interpretation and classified to identify land cover types. These preprocessing steps ensure the accuracy and applicability of the data. The data on green spaces and rural residential areas comes from Amap Application Programming Interface (API) on Amap Maps, collected in June 2022. This data contains basic information about rural communities and park green spaces, such as name, type, area, and latitude and longitude coordinates. These data help analyze the spatial layout of human activities within cultural heritage conservation areas and their relationship with the surrounding environment. To ensure the validity of the data, all collected records are cleaned, invalid or incomplete entries are removed, and the data is converted to a format compatible with other datasets. Meanwhile, the data is geo-coded to ensure location accuracy. The population distribution data was collected from public websites through a web crawler program written in Python from July to September 2022, with a spatial resolution of 25 meters. The data is sourced from the National Bureau of Statistics’ public data platform [40]: http://www.stats.gov.cn; Baidu Maps Heat Map Data Interface [41]: https://lbsyun.baidu.com; Gaode Map Open Platform [42]: https://lbs.amap.com. This data provides the population numbers of various regions within the area, including latitude, longitude, and calorific value (i.e., relative population). These data play a crucial role in analyzing the accessibility of residents and the socio-economic impact of cultural heritage areas. In order to eliminate redundancy and outliers in the data, all data is cleaned and standardized to convert population size into relative heat values, providing a fair comparison basis for subsequent spatial analysis. Table 1 exhibits specific details.

**Table 1:** Data types and sources.

Data types	Sources	Data collection time	Spatial resolution	Attribute information	Preprocessing plan
High-resolution satellite image data [43]	Resources satellite three (ZY-3), Gaofen-1 satellite (GF-1), Gaofen-2 satellite (GF-2)	February 2018	–	Image data are used for surface feature extraction and visualization.	Image preprocessing measures such as radiometric correction, atmospheric correction, and geometric correction are conducted to ensure the quality of image data. Surface features are extracted through image interpretation to identify and classify different types of land cover.
Green spaces and rural settlement data [44]	Amap Application Programming Interface (API) for application development	June 2022	–	This includes basic data such as the name, type, area, and latitude-longitude coordinates of rural communities/park green spaces.	The data is cleaned to remove invalid or incomplete records, converted to a format compatible with other datasets, and geocoded to ensure the accurate location of the settlement data.
Population distribution data [40–42]	Python-written web scraping program	July to September 2022	25m	This includes fields such as data acquisition time, longitude, latitude, and heat values to represent the relative population quantities.	The data is cleaned to remove duplicate records, and handle missing values and outliers. Then, the data is standardized and the population size is converted into relative calorific values for spatial analysis.

Table 1 utilizes high-resolution satellite image data to capture surface features, as this type of data provides detailed land cover information that aids in identifying natural landscapes and man-made structures. Additionally, green space and rural settlement data obtained through the Amap API exist in the form of points and polygons, offering a direct view of the spatial structure and functional distribution within the cultural heritage area. Population distribution data, collected in the form of point data through web scraping programs, reflect the demographic dynamics of the area, which is crucial for analyzing the accessibility and socio-economic impact of cultural heritage. The selection of these datasets is based on their ability to comprehensively reflect the multi-dimensional characteristics of the cultural heritage area, including the natural environment, human activities, and cultural assets. The satellite image data covers different periods, ensuring that the analysis takes into account the effects of seasonal variations; Settlement data provides an in-depth understanding of actual spatial usage and population distribution. Through the integrated analysis of these data, the spatial distribution and accessibility of the cultural heritage area can be more accurately assessed, offering solid data support for conservation strategies and urban planning. For high-resolution satellite image data, radiation correction, atmospheric correction, geometric correction, and other image preprocessing measures are firstly carried out to ensure the quality of image data. Secondly, surface features are extracted through image interpretation to identify and classify different land cover types. For green space and rural settlement data, the data obtained from the Amap API is cleaned to remove invalid or incomplete records. In addition, the data format is converted to make it compatible with other datasets and geocoded to ensure that the location of the settlement data is accurate. For the population distribution data, cleaning measures such as removing duplicate records, and processing missing values and outliers are first carried out. Then, the data is standardized to convert the population number into relative calorific values for use in spatial analysis. This allows for a more equitable comparison between different regions and helps to reveal the relative density of population distribution. Lastly, data from different sources may have varying spatial resolutions (some data may be in meters while others may be in kilometers) and scales (some maps may be more detailed while others may be more generalized). These data must be transformed into a unified spatial resolution and scale to compare and integrate data within the same analytical framework. Spatial interpolation or resampling techniques can be used to adjust the spatial resolution of the data, ensuring consistency across all datasets. This involves downsampling high-resolution data to a lower resolution or upsampling low-resolution data to a higher resolution. For resampling or aggregating data, it should conform to the spatial scale required for the study. Of course, when collecting and using cultural heritage data, it is essential to strictly adhere to ethical and privacy guidelines, respect the confidentiality and sensitivity of cultural heritage, and ensure the legal acquisition and use of data.

### Experimental evaluation

This study focuses on the Weibei Imperial Mausoleum Protection Zone as the research area to evaluate the spatial distribution performance of the constructed settlement spatial distribution model based on factor analysis integrated with the hierarchical clustering algorithm. Data sources include three types of data: satellite high-resolution image data, green space and rural settlement data, and population distribution data, as detailed in Table 1 above. First, spatial distribution classification is conducted at six levels: distribution, density, land use, scale, shape, and cultural heritage. This study constructs a settlement spatial distribution model based on a hierarchical clustering algorithm and factor analysis. The proposed model examines the relationship between the number of classifications, the GVF, and the Sum of the Squared Errors (SSE) for each combination. Here, the number of classifications refers to the division of data points into how many distinct categories or groups in hierarchical cluster analysis. This indicator is a key decision point in cluster analysis as it directly affects the interpretation and application of the clustering results. In this study, the selection of the number of classifications is based on the understanding of the data structure and the evaluation of cluster quality indicators. The GVF is a statistical measure used to assess the goodness of fit of hierarchical clustering algorithms in spatial distribution analysis. A higher GVF value indicates better clustering results, meaning that the variance within clusters is smaller and the variance between clusters is larger after clustering. It suggests that the clustering reveals the inherent structure of the data. This study employs GVF to evaluate the effectiveness of hierarchical clustering under different numbers of classifications. The SSE is another important indicator for measuring cluster quality, which calculates the sum of the squared Euclidean distances between each data point and the centroid of its cluster. A lower SSE value illustrates that the data points within a cluster are more tightly clustered around the centroid, implying better compactness of the cluster. When selecting the optimal number of classifications, one usually looks for the point where the SSE value begins to decrease significantly, which typically suggests that adding more classifications does not substantially improve cluster quality. Furthermore, the proposed model algorithm is compared with K-means [45], Kernel Density Estimation (KDE) [46], and the algorithm proposed by Zerouali et al. (2022) from the perspective of silhouette coefficient to analyze the spatial distribution characteristics in the research area.

Of these, six levels are detailed as follows. First, the distribution level focuses on the geographical distribution of settlements throughout the study area, including how they are dispersed or clustered. Second, the density level examines the number of settlements per unit area, that is, the spatial density of settlements. Third, the land use level analyzes the relationship between land use types and settlement distribution and discusses how different types of land use affect the spatial layout of settlements. Fourth, the scale level looks at the size or scale of the settlement, which may include the area occupied by the settlement, the number of people living in it, and so on. Fifth, the shape level evaluates the shape characteristics of settlements, such as whether they exhibit regular or irregular shapes, and how these shape characteristics fit into the surrounding environment. Sixth, the cultural heritage level concentrates on the distribution of cultural heritage, covering the density and diversity of cultural heritage sites and their relationship to surrounding settlements.

Next, the overall and local accessibility of tourists and the public to the Weibei Imperial Mausoleum Protection Zone are visually and effectively compared. Leveraging GIS for spatial analysis, population distribution data is overlaid with the spatial distribution of cultural heritage sites to identify the correlation between population density and cultural heritage accessibility. Meanwhile, this study utilizes a Natural Breaks method to classify comprehensive accessibility on weekdays and weekends. This classification comprises six levels, and the breakpoint values are taken as the criteria for the accessibility levels, as outlined in Table 2.

**Table 2:** Classification of accessibility levels.

	Accessibility levels					
	Lowest	Medium-low	Lower	Higher	Medium-high	Highest
Weekdays	<2	2–8	8–13	13–24	24–50	>50
Weekends	<2	2–7	7–12	12–25	25–50	>50

Table 2 demonstrates the classification of accessibility levels in the Weibei Imperial Mausoleum Protection Zone, determined by considering the convenience for residents and tourists to visit cultural heritage sites on weekdays and weekends. The Natural Breaks (Jenks) method is used to categorize accessibility levels, which can identify natural clusters in the data, maintaining similarity within each category while preserving differences between categories. Specifically, accessibility is divided into six levels—lowest, medium-low, low, medium, medium-high, and highest—based on the average time it takes for residents to reach cultural heritage sites. For instance, on weekdays, if the average time for residents to reach the nearest cultural heritage site is less than 2 minutes, the area is classified as the “lowest” accessibility level; if the average time is between 2 and 8 minutes, it is classified as the “medium-low” level, and so on. This grading method helps to understand the changes in the accessibility of cultural heritage sites at different times more delicately, providing decision support for urban planning and cultural heritage protection. This classification identifies which areas are likely to face visitation pressure during specific periods, allowing for measures to optimize traffic flow and enhance the tourist experience.

## Results and discussion

This section focuses on the spatial distribution characteristics and accessibility analysis results of settlements in the Weibei Imperial Mausoleum Protection Zone. By combining the hierarchical clustering algorithm and factor analysis, a reasonable number of classifications is determined, and the model’s accuracy is verified by GVF and average silhouette coefficient. In addition, the pattern of residents visiting cultural heritage sites in different periods is analyzed, offering data support and strategic suggestions for urban planning and cultural heritage protection.

### Result analysis of spatial distribution characteristics

**Fig 4:**
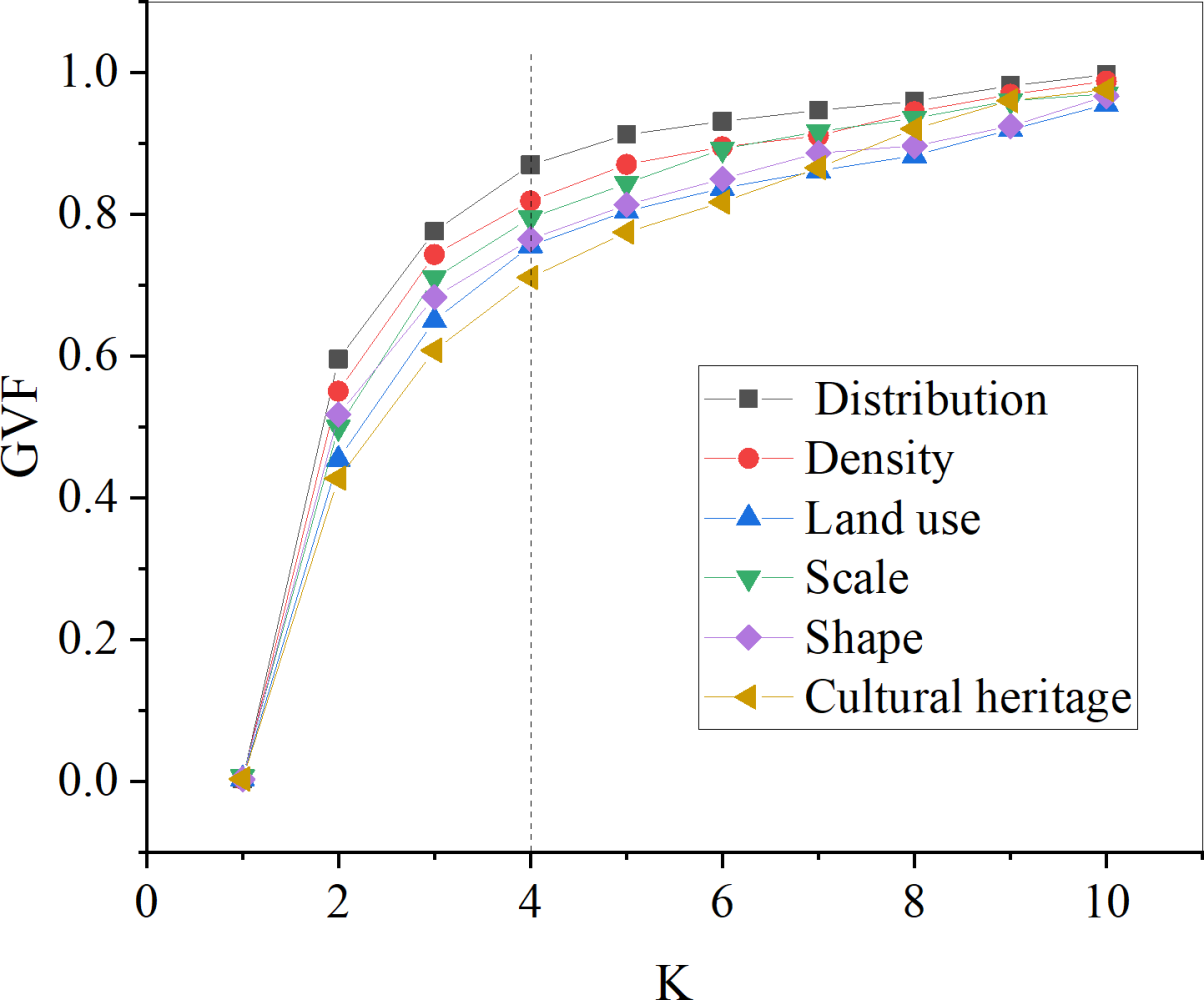
The correlation results graph between the number of classifications (K) and the GVF for each combination.

**Fig 5:**
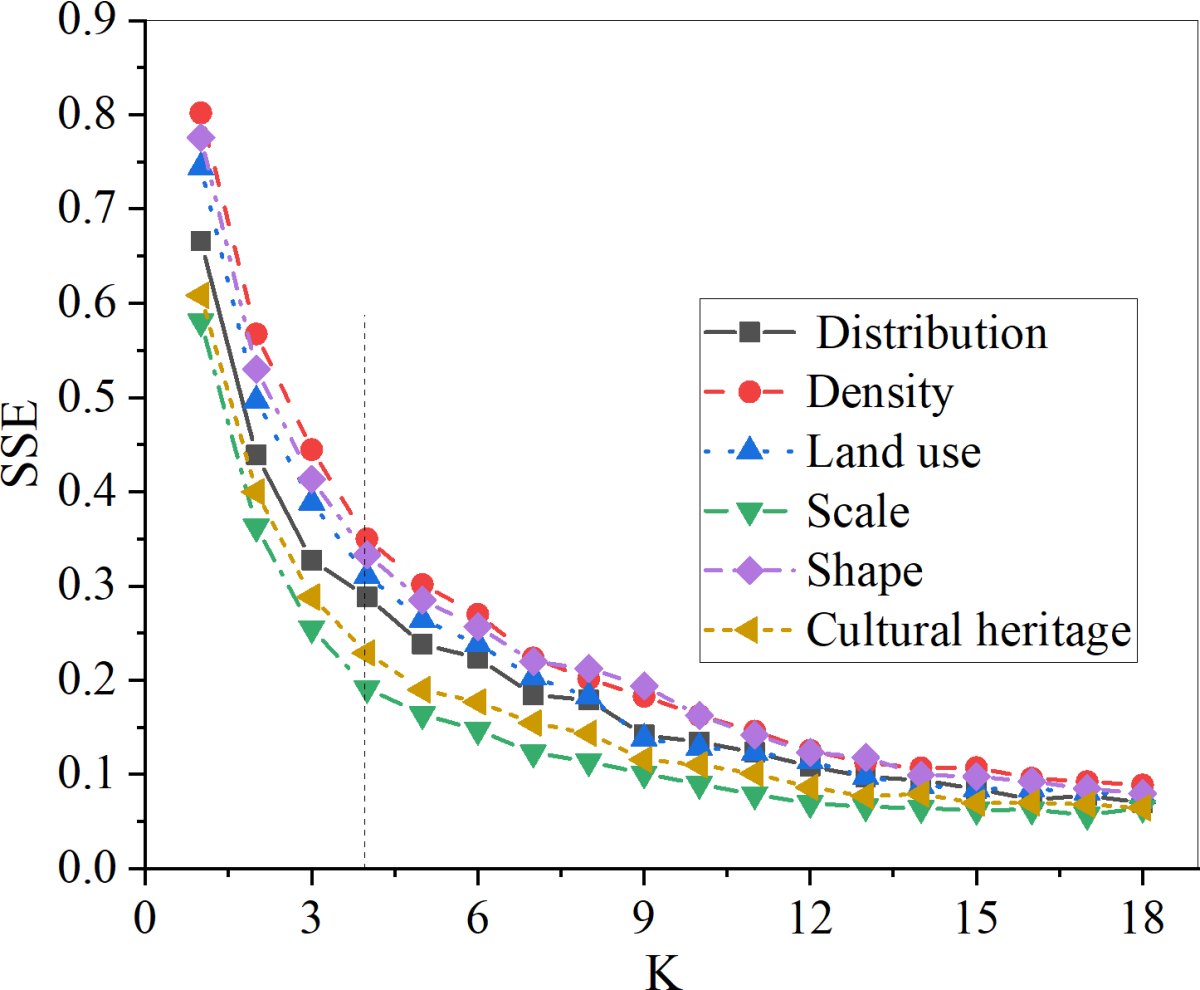
The relationship between the number of classifications (K) and the SSE results graph.

The spatial distribution characteristics analysis assesses the relationship between the number of classifications (K) and the GVF and SSE for each combination across six levels. They are distribution, density, land use, scale, shape, and cultural heritage. Figs 4 and 5 display the results.

Fig 4 reveals that the GVF curves for the six-level features exhibit similar trends. As K increases, the curves become increasingly flat. When K is greater than 4, the growth rate of GVF for each feature decreases; at this point, all GVF values exceed 0.7. Particularly for distribution and density, the flattening trend of the curves is significant, and when K is higher than 4, GVF values are above 0.8, illustrating a better classification performance. Therefore, dividing each level into four categories is a reasonable choice.

In Fig 5, the SSE for each level is analyzed. It indicates that the SSE curves for the six-level features exhibit similar trends. As K increases, the SSE values first decrease and then become increasingly flat. When K is greater than 4, the descent rate of SSE for each feature presents a declining trend, especially for scale and historical heritage. Therefore, determining K as 4 can result in a better classification performance, which aligns with the expected subset partition.

Lastly, spatial distribution classification is performed across six levels: distribution, density, land use, scale, shape, and cultural heritage. Table 3 presents the four categories for each level.

**Table 3:** Spatial distribution level results.

Layers	Types	Classifications			
The first layer	Distribution	Highly fragmented and uniform type	Moderate fragmentation and dispersion type	Settlement type	Aggregated type
The second layer	Density	Low-density type	Moderate-low density type	Moderate-high density type	High-density type
The third layer	Land use	Low-diversity type	Moderate-low diversity type	Moderate-high diversity type	High-diversity type
The fourth layer	Scale	Large-scale village type	Moderate-large-scale village type	Moderate-small-scale village type	Small-scale village type
The fifth layer	Shape	Type with highly diverse settlement shape	Type with moderate diverse settlement shape	Type with slightly diverse settlement shape	Type with slightly diverse settlement shape
The sixth layer	Cultural heritage	Type with intensive cultural heritage	Types rich in cultural heritage	Types with few cultural heritage	Type with scattered cultural heritage

**Fig 6:**
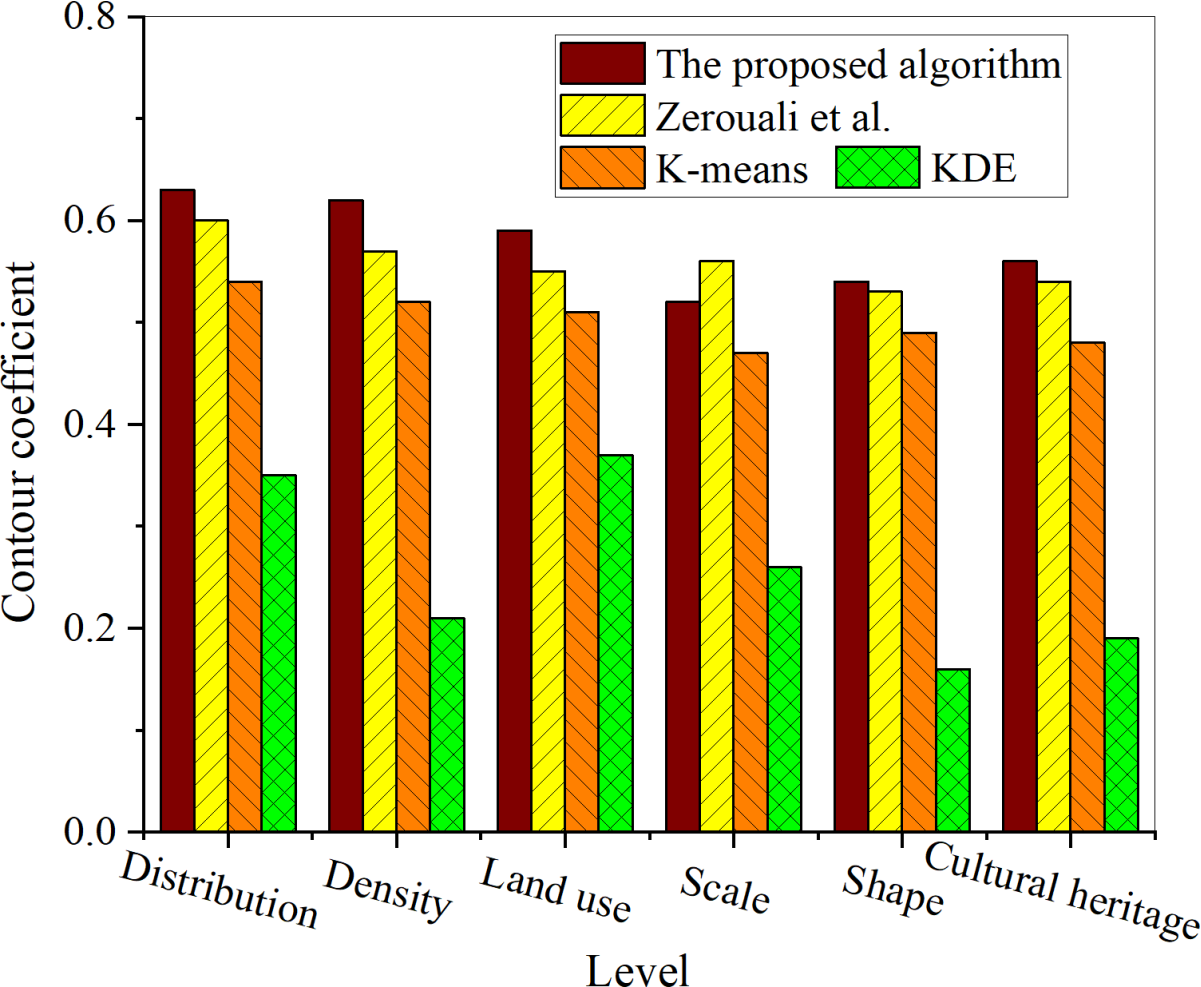
The silhouette coefficient scores for different algorithms at various levels.

Fig 6 lists the silhouette coefficients for each algorithm at different levels when the number of classifications is 4.

Fig 6 shows that compared to other algorithms, the proposed algorithm’s silhouette coefficient is higher, with an average silhouette coefficient reaching 0.58, while the highest silhouette coefficient for KDE is only 0.37. A further analysis of the silhouette coefficients at different levels reveals that the distribution level has the highest silhouette coefficient, reaching 0.63. The higher the silhouette coefficient, the more similar the samples within a cluster, which means that the points within the same cluster are more closely clustered. In the analysis of spatial distribution characteristics, this may imply that neighboring locations have similar features, which could be due to shared geographic environments, land use, or resource distribution, among other factors. Hence, when the number of classifications is set to 4, the spatial distribution characteristics can be better identified to support the spatial layout optimization of cultural heritage areas.

### Accessibility results analysis

**Fig 7:**
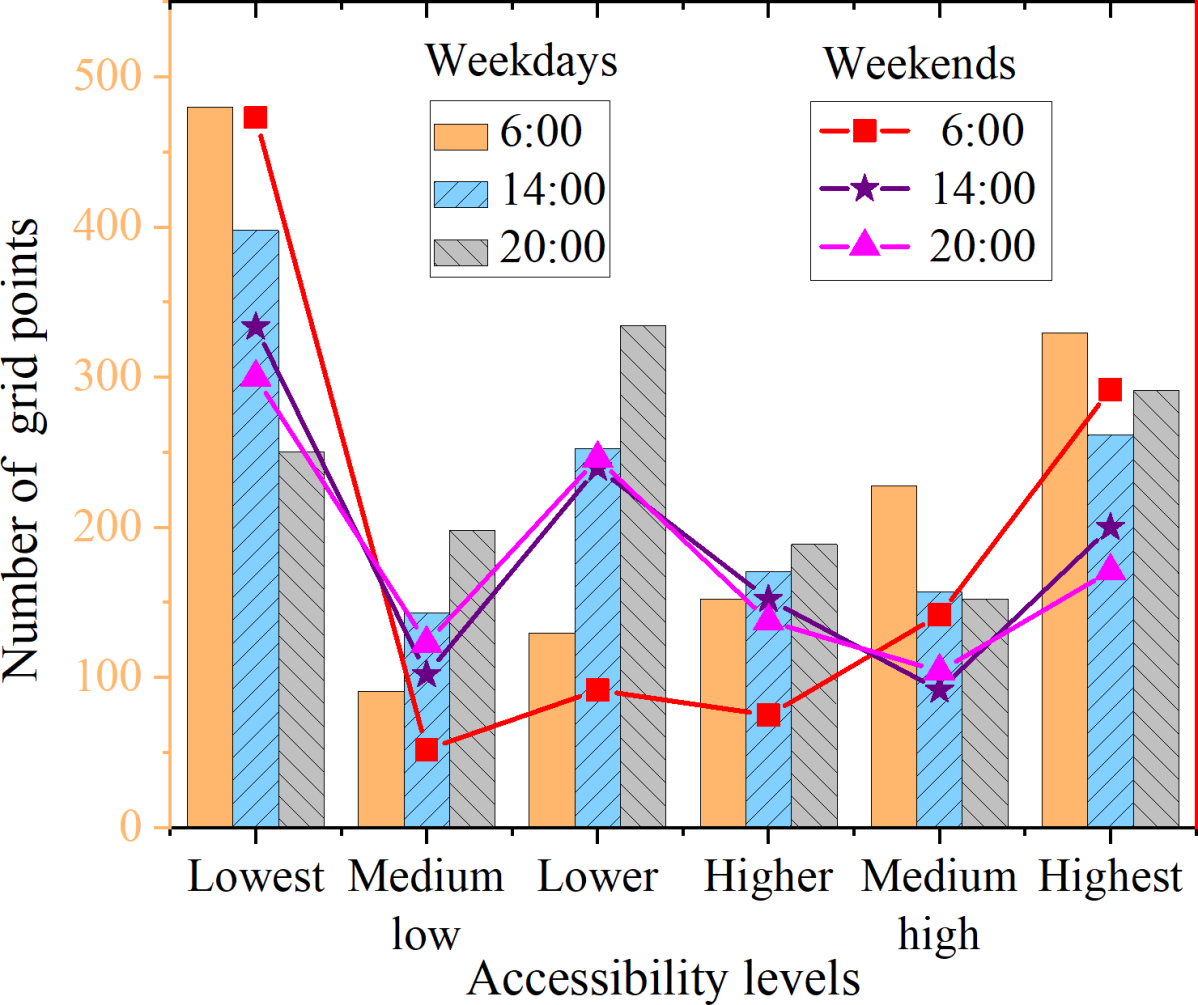
Comparison of grid points for each accessibility level at different time points on weekdays and weekends.

**Fig 8:**
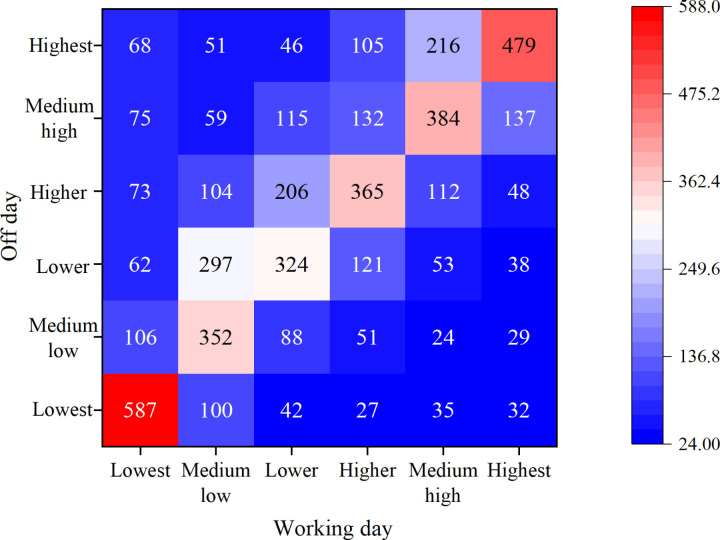
The transition matrix chart for comprehensive accessibility levels on weekdays and weekends.

The comprehensive accessibility characteristics of the Weibei Imperial Mausoleum Protection Zone are analyzed at various time points (6:00 AM, 2:00 PM, 8:00 PM) and across two-time scales: weekdays and weekends. Figs 7 and 8 depict the results.

Fig 7 suggests that the trends in the number of grid points for accessibility levels on weekdays and weekends are quite similar. Specifically, at 6:00 AM, the number of grid points in the low accessibility level on weekends slightly exceeds that on weekdays. However, the grid points for the medium-low, lower, and higher accessibility levels remain relatively low until the appearance of the medium-high and high accessibility levels. In contrast, the trends at 2:00 PM and 8:00 PM are similar, with the number of grid points in the low accessibility level on weekdays being higher than on weekends. The medium-low accessibility level experiences a significant decrease, followed by an increase in the low accessibility level. Then, a decrease again in the higher and medium-high accessibility levels until there is a subsequent increase in the high accessibility level. The result analysis indicates that at 6:00 AM, there are more grid points in the low and high accessibility levels, while the medium levels have fewer grid points. At 2:00 PM and 8:00 PM, which are similar, there are more grid points in the medium levels. At these time points, the increase in the population of the area leads to increased difficulty in accessing heritage conservation park resources, resulting in reduced accessibility.

Fig 8 underscores that in the transition from weekdays to weekends, 100 grid points have upgraded from “lowest” to “medium-low,” illustrating that people find it easier to access the heritage protection zone on weekends. Conversely, in the transition from weekends to weekdays, 112 grid points have descended from “higher” to “medium-high,” possibly due to weekday traffic and crowd impacts on accessibility. The stability within the same level is reflected in the relative consistency of accessibility between weekdays and weekends. Therefore, through this transition matrix, insights into residents’ behavioral patterns at diverse time points can be gleaned, which can be used for urban planning and green space management to meet residents’ needs.

### Discussion

This study indicates that the hierarchical cluster analysis of the proposed model algorithm achieves a GVF value exceeding 0.7 when the number of classifications is set to 4. Additionally, the average silhouette coefficient reaches 0.58, a value higher than the corresponding indicators in studies by Su et al. and Wu et al., indicating that the clustering results have greater internal consistency and discriminability [47,48]. These results not only confirm the rationality of the clustering outcomes but also reflect the precision of this study in revealing the spatial distribution patterns of cultural heritage sites. In terms of accessibility analysis, this study categorizes comprehensive accessibility into six levels using the Natural Breaks method and compares them across different periods on weekdays and weekends. For instance, on weekends, 100 grid points are upgraded from the “lowest” to “medium-low” accessibility level, while 112 grid points are downgraded from “high” to “medium-high” level from weekdays to weekends. These specific figures reveal the behavioral patterns of residents accessing cultural heritage sites at different times, echoing the findings of Yang & Li on the impact of temporal factors on accessibility [49]. This study’s strength lies in its provision of more nuanced and dynamic decision support by integrating both temporal and spatial factors, which holds significant practical value in the practice of cultural heritage conservation and urban planning.

In this study, the impact of land use types on the accessibility of cultural heritage sites is also taken into consideration. Land use not only determines the environmental characteristics surrounding cultural heritage sites but also directly affects traffic flow and tourist access patterns. For example, commercial and entertainment land uses may attract more tourists on weekends and holidays, thereby affecting the accessibility of cultural heritage sites. Additionally, the distribution of residential land use can influence the daily access of residents to cultural heritage sites. The analysis shows that the diversity and distribution of land use significantly impact the accessibility of cultural heritage sites, which is particularly evident when comparing weekdays and weekends. Therefore, future urban planning and cultural heritage conservation strategies should pay more attention to land use planning to optimize the accessibility of cultural heritage sites.

The shape and size of settlements are another critical factor affecting the accessibility of cultural heritage sites. Larger settlements may offer more services and facilities, attracting tourists and residents, but may also lead to traffic congestion, especially during peak hours. In contrast, smaller settlements may provide a quieter visiting experience but may lack necessary services and facilities. The study’s results indicate that the scale and shape of settlements have a complex impact on the accessibility of cultural heritage sites, which requires careful consideration in urban planning and cultural heritage conservation. For instance, by improving transportation connections and providing necessary service facilities, the attractiveness and accessibility of cultural heritage sites in smaller settlements can be enhanced.

In addition to time and date, other parameters such as land use, and the shape and size of settlements also demonstrate remarkable contributions to the accessibility of cultural heritage sites in this study. These parameters not only affect the accessibility of cultural heritage sites but also influence the perceptions and experiences of tourists and residents. For instance, a settlement with a unique shape and historical value may attract more tourists, even if its physical accessibility is not ideal. Therefore, future research can further explore how these parameters interact with each other and how they collectively impact the accessibility and sustainability of cultural heritage sites.

The study reveals the spatial distribution characteristics of cultural heritage sites within the Weibei Imperial Mausoleum Protection Zone. Meanwhile, it assesses accessibility during different periods, providing new strategies for the conservation and management of cultural heritage. Firstly, cultural heritage sites can be classified and managed based on their spatial distribution characteristics to allocate conservation resources and develop targeted conservation measures more effectively. For example, those cultural heritage sites that show high tourist traffic in cluster analysis may require more protection and maintenance resources, as well as stricter crowd control measures. Secondly, by analyzing accessibility during diverse periods, this study offers a basis for dynamically adjusting the opening hours of cultural heritage sites and tourist guidance strategies. Thus, it helps to balance the tourist experience with the conservation needs of cultural heritage.

Furthermore, the study’s comprehensive consideration of various spatial and temporal factors represents an important advancement in the practice of cultural heritage conservation and urban planning. Through this approach, a more comprehensive understanding of the usage and conservation needs of cultural heritage sites can be achieved, filling the gaps in existing research and practices. Especially when dealing with large-scale and multi-scale spatial distribution data, this study provides an effective analytical framework, a valuable tool for decision-makers in cultural heritage conservation and urban planning. Consequently, this study not only offers specific management strategies for the Weibei Imperial Mausoleum Protection Zone but also provides a replicable approach for the conservation and management of other cultural heritage sites.

## Conclusion

This study uses a combination of the hierarchical clustering algorithm and factor analysis to conduct an in-depth analysis of the spatial distribution characteristics of settlements in the Weibei Imperial Mausoleum Protection Zone and a comprehensive assessment of their accessibility. The findings of this study reveal that the spatial distribution of settlements in the Weibei Imperial Mausoleum Protection Zone has obvious hierarchy and aggregation. Through factor analysis and dimensionality reduction, the key factors affecting settlement distribution are successfully extracted, and the settlements are divided into four categories by the hierarchical clustering algorithm. The GVF value surpasses 0.7 and the average silhouette coefficient reaches 0.58, indicating that the classification results are reasonable and have high consistency. In terms of accessibility, it is found that accessibility presents apparent time dependence at different time points, and accessibility is somewhat reduced during the morning and evening peak periods due to the influence of traffic flow and the number of tourists.

Meanwhile, the results of this study are of great significance for understanding and optimizing the cultural heritage protection and urban planning of the Weibei Imperial Mausoleum Protection Zone. By revealing the spatial distribution patterns of settlements and the accessibility of cultural heritage sites, this study provides scientific decision support for relevant decision-makers. This study not only offers a scientific basis for the urban planning and cultural heritage protection of the Weibei Imperial Mausoleum Protection Zone but also provides a methodological reference for the spatial distribution and accessibility research of other similar cultural heritage areas. Regarding policy recommendations, it is suggested that policy-makers and urban planners should fully consider the spatial distribution characteristics and accessibility of cultural heritage sites when formulating relevant policies and management measures, to promote the protection and sustainable development of cultural heritage.

Although this study has achieved certain results, there are also some limitations. For example, data is collected over a short period and may not fully reflect long-term trends. Future studies could consider longer datasets to explore dynamic changes on different time scales. In addition, this study mainly focuses on the spatial distribution of settlements and accessibility. Future research can be extended to analyzing other cultural heritage elements within the protected area. Of course, it is also possible to consider applying the research method to the spatial distribution analysis of other cultural sites to verify its universality.

## Supporting information

S1 File The relevant data and code in the manuscript can be found in the supporting information “Data&Code” file.
(ZIP)
