# Supplementary material for: Spatial distribution characteristics and accessibility analysis in the Weibei Imperial Mausoleum Protection Zone under the hierarchical clustering algorithm
Source: PLoS One. 2025 May 8;20(5):e0321847. doi: 10.1371/journal.pone.0321847 (PMC12061419; doi:10.1371/journal.pone.0321847)
Supplement: S1 File — The relevant data and code in the manuscript can be found in the supporting information “Data&Code” file. (ZIP) [file pone.0321847.s001.zip › Code description and configuration.docx]

**Code Description**

1. **Import necessary libraries:**
   - numpy: It is used for mathematical operations and array operations.
   - FactorAnalysis from sklearn.decomposition: It is utilized to perform factor analysis and reduce data dimensionality.
   - pdist and squareform from scipy.spatial.distance: They calculate the distance between samples.
   - linkage and fcluster from scipy.cluster.hierarchy: They are employed to perform hierarchical clustering and assign clustering labels from scipy.cluster. hierarchy.
2. **Factor analysis function** (factor_analysis):
   - Input: raw data and the number of components to be extracted.
   - Processing: It uses the FactorAnalysis class to perform dimensionality reduction on the data.
   - Output: Reduced dimensional data.
3. **CURE clustering function** (cure_clustering):
   - Input: Reduced dimensional data, number of clusters, and number of representative points for each cluster.
   - Processing: It calculates the distance matrix between all samples, performs hierarchical clustering using the average linking method, and randomly selects representative points for each cluster.
   - Output: Cluster labels for each sample and representative points for each cluster.
4. **Integrated model function** (integrated_model):
   - Input: the number of components in raw data, factor analysis, and CURE clustering.
   - Processing:
     - First, factor analysis is used to reduce the dimensionality of the data.
     - Second, the CURE clustering algorithm is applied to the reduced dimensional data.
   - Output: Cluster labels for each sample and representative points for each cluster.

**Configuration Description**

- **Data input:** The data variable in the code should be a two-dimensional array, where rows represent samples and columns represent features.
- **Factor analysis parameters:**
  - n_components: It specifies the number of components to be extracted in factor analysis, depending on the characteristics of the data and the analysis objectives.
- **CURE clustering parameters:**
  - num_clusters: It specifies the number of clusters to be formed.
  - num_rep_points: It specifies the number of representative points to be selected in each cluster, which helps capture the shape and size of the cluster.
- **Output:**
  - cluster_labels: A one-dimensional array containing the clustering labels for each sample.
  - representative_points: A two-dimensional array containing representative points for each cluster.
